# A Review of the Management of Chronic Scrotal Pain

**DOI:** 10.7759/cureus.11979

**Published:** 2020-12-08

**Authors:** Amr Moubasher, Muhammad Waqar, Nicholas Raison, Oliver Brunckhorst, Kamran Ahmed

**Affiliations:** 1 Urology, King's College Hospital, London, GBR; 2 Dermatology and Andrology, Assiut University, Assiut, EGY; 3 Urology, MRC Centre for Transplantation, Guy's Hospital Campus, King's College London, King's Health Partners, London, GBR

**Keywords:** scrotal pain, testicular pain, orchialgia, clinical evaluation, diagnostic modalities, medical treatment, surgical treatment

## Abstract

Chronic scrotal pain (CSP) is a common and poorly understood medical condition that significantly affects individuals’ quality of life. Many patients seek evaluation and management of their symptoms from multiple physicians. Our review aims to address diagnostic modalities, clinical evaluation, and surgical and non-surgical management.

We conducted a computerised detailed search of the PubMed, Medline, Embase and Cochrane databases for reports pertaining to CSP using the Medical Subject Headings keywords ‘chronic scrotal pain’, ‘testicular pain’ and ‘orchialgia’, and we included in the review those that fulfilled the inclusion (adult male with CSP presenting with the criteria of CSP ) and exclusion (extra-scrotal pain) criteria.

After the direct causes of CSP were identified by reviewing the clinical evaluations (history taking and examination are mandatory) and the diagnostic evaluations (urine analysis is crucial and ultrasound can be helpful), the most-used medical and non-surgical treatments for CSP were tricyclic antidepressants (success rate of up to 66.6%) and spermatic block (success rate of more than 90%), and the most-used surgical procedure was microsurgical denervation of the spermatic cord (success rate of up to 70%).

The evidence currently available remains rare and of low quality, making it difficult to strongly recommend individual treatment options. However, multimodal treatment modalities using physical therapy and psychotherapy may help patients and provide useful tools for coping with this condition. There are also useful non-surgical and surgical options for CSP that depend on the patient’s state, the severity of the complaint and what options have already been tried.

## Introduction and background

Scrotal pain, in both its acute and chronic forms, is a diagnostic challenge that must be carefully evaluated using a full patient history and physical examination. However, a complete examination may be precluded by pain, the dependent nature of the scrotum, and the oedema and skin changes accompanying many scrotal pathologies. The physical examination must also include a careful evaluation of the abdomen and inguinal region and a genital examination to assess possible herniation [1]. Knowledge of the patient’s age, sexual history, and the duration, severity and onset (gradual vs. sudden) of the pain is necessary to focus the clinician’s attention on the correct diagnostic path.

Chronic scrotal pain (CSP) is defined as at least three months of chronic or intermittent scrotal content pain of a severity that interferes with daily activities and prompts the patient to seek medical advice [2]. CSP may originate from the testicle, epididymis, para-testicular structures and/or the spermatic cord. The aetiology of the pain is unknown in up to 50% of patients [3]. The recognisable and reversible causes of CSP include varicocele, epididymitis, spermatocele, tumour, infection and torsion. This condition has been referred to by many names, including chronic orchialgia, testicular pain syndrome, testalgia, chronic scrotal content pain, post-vasectomy orchialgia, post-vasectomy pain syndrome (PVPS), congestive epididymitis and chronic testicular pain. To date, there are no definitive data on the incidence and prevalence of this condition in the general population. Several studies have provided estimates of the prevalence of the condition in patients, with frequencies ranging from 0.4% to 4.75% in specific groups of men [4].

Multiple algorithms for diagnosing and treating CSP have been proposed, but none have been validated [5,6] because it is a difficult condition to manage, and varied practices exist with reference to it. Therefore, our review aimed to:

1) Overview current approaches to the clinical evaluation of CSP.

2) Overview currently used diagnostic modalities.

3) Discuss the efficacy of non-surgical management options for CSP.

4) Review the current options for surgical management of CSP.

## Review

Method

We conducted a computerised bibliographic search of the PubMed, Medline, Embase and Cochrane databases for all reports pertaining to CSP using the Medical Subject Headings keywords ‘chronic scrotal pain’, ‘testicular pain’ and ‘orchialgia’. The search found 292 studies related to CSP, 339 related to chronic testicular pain and 22 related to chronic orchialgia. Because all 653 studies met the criteria for inclusion, all were included in the review. The inclusion criteria included adult males having CSP and presenting with the criteria for CSP, and the exclusion criteria included extra-scrotal pain.

Results

Clinical Evaluation

Most of the reviewed publications agreed regarding the points for taking histories and making examinations. One Turkish study involved men who had presented to outpatient urology clinics [7,8]. Questionnaires had been used to diagnose 4.75% of these men as having CSP. Another study surveyed urologists in Switzerland, who reported that 2.5% of consultations with male patients were due to CSP [9,10]. Still, another study from Israel had assessed all young men presenting for military service and had found that 0.8% of them had been examined by a physician for CSP [11]. Although these studies suggest that CSP is a common condition, further studies are needed to confirm the true incidence and prevalence of the condition in the general population. Certain groups of men have a higher incidence of CSP: (1) post-vasectomy pain affects from 1% to 15% of men after their procedures [12,13], (2) an estimated 10% of men with varicocele experience CSP related to that condition [14] and (3) CSP has been reported in as many as 3% to 6% of patients after repair of inguinal hernia [15].

CSP significantly affects one’s quality of life. The condition can significantly limit daily activities and adversely affect occupational and social functioning. History points crucial to diagnosis include pain location, subjective description (sharp, dull, burning), timing (onset, duration, constant vs. intermittent), radiation to surrounding structures and severity (we used a Likert-type visual analogue pain scale that ranges from 0 to 10). Other questions to ask include: Are there precipitating factors such as activity or positional changes (ambulation or prolonged sitting)? Does the pain change with urination, defecation or ejaculation? Is there scrotal swelling or a mass? The answers to these questions reveal information that is vitally important to concluding the differential diagnosis [5].

Prior abdominal or pelvic surgeries, including inguinal hernia repair, varicocelectomy and surgery for undescended testicle, and prior spinal or orthopaedic surgeries provide answers regarding the presence of anatomic contributions to CSP. The content of a detailed social history also informs diagnosis and treatment. For instance, a history of sexual abuse has been associated with increased rates of chronic pelvic pain in adulthood and may affect subsequent approaches to physical examination and adjunctive testing [16]. Up to 40% of patients who have CSP complain of depressive symptoms, and many feel socially isolated [17,18]. CSP patients often complain of anxiety related to concerns about testicular cancer. Early assessment of these concerns helps to create a partnership between the provider and patient [19]. The medical history should include prior sexually transmitted diseases, childhood urologic conditions, back and spinal pathologies and any underlying psychological conditions including anxiety and depression [20].

A complete physical examination of the abdomen and genitalia is mandatory, and clear communication during this examination is imperative. A visual inspection of the scrotum and inguinal region will reveal any scars from prior surgeries or traumas. Swelling may include a solid mass, hydrocele or varicocele. Careful palpation of the testicles, epididymis, vas deferens and spermatic cord enables comparison of focal and more-diffuse tenderness. In cases of prior vasectomy, fullness or overt swelling of the epididymis and testicle and pain over the sperm granuloma are often visualised. Importantly, a digital rectal examination (DRE) that includes careful palpation of the entire rectal vault, prostate and pelvic floor musculature should be performed routinely, as it can significantly alter treatment recommendations. Positive focal or diffuse tenderness involving the prostate or pelvic floor musculature as well as a generalised increase in anal/rectal tone suggest precipitating pelvic floor tension myalgia or chronic prostatitis [6].

Diagnostic Modalities

As mentioned, most of the publications reviewed agree on the points regarding taking histories and conducting examinations. In addition, adjunctive testing supplements these histories and physical examinations. The initial evaluation should include urinalysis with microscopy to exclude haematuria or pyuria, both of which can be seen in cases of infectious aetiologies or referred pain from urolithiasis. If microscopic haematuria is identified, further workup includes cross-sectional imaging of the abdomen/pelvis and possibly cystoscopy [7]. Scrotal ultrasonography identifies structural causes within the scrotum, including tumours, cysts or subclinical varicocele, and is recommended in most circumstances. Imaging of the spine or hips is indicated if symptoms suggest any underlying involvement of the musculoskeletal system. In the case of new-onset varicocele, particularly on the right side, cross-sectional imaging of the abdomen and pelvis must be considered to rule out a retroperitoneal or abdominal process [8].

Non-surgical management

Medical Treatment

The treatment of CSP suffers from a lack of evidence-based references. Although clinical data exist, most reports in the literature regarding treatment consist of case series without control groups [21,22]. Conservative management is helpful after ruling out any underlying structural cause (i.e., scrotal mass, varicocele or inguinal hernia) or a source of referred pain (i.e., ureteral calculus, hip or labrum disease or spinal pathology) [23].

If a urinary tract or other genital infection is present, it should be treated accordingly. Antimicrobial therapies for epididymitis are dependent on age and other risk factors [24,25]. According to the literature, fluoroquinolones and trimethoprim-sulfamethoxazole are the antibiotics most used, and these are the initial non-surgical treatments for these patients [26,27]. The second most commonly mentioned option for idiopathic CSP is non-steroidal anti-inflammatory drugs (NSAIDs), which are appealing given their ability to address pain and inflammation [28,29]. On occasion, this may be the only therapy necessary.

The most used non-surgical options for idiopathic CSP are tricyclic antidepressants (TCAs), anticonvulsants and spermatic cord blocks (SCBs). TCAs have a success rate of 66.6% for idiopathic testicular pain, and anticonvulsants have an improvement rate of 61.5% [30,31].

TCAs act by inhibiting the reuptake of norepinephrine and serotonin in the brain. They also inhibit sodium channel blockers and L-type calcium channels, and this is thought to be the cause of their analgesic effect: they modulate first-order neuron synapses with second-order ones in the dorsal horn of the spinal cord [28,29]. Tertiary amines (amitriptyline and clomipramine) have been reported as more effective for neuropathic pain than secondary amines (desipramine and nortriptyline) [30,31]. However, tertiary amines are also accompanied by more sedation and postural hypotension [32,33]. A TCA may take two to three weeks from initiation of therapy to have an effect. It is crucial to taper patients off TCAs [32,33].

In one case, after a month of unsuccessful TCA therapy, it was agreed to add an anticonvulsant, as they have been shown to influence neuropathic pain. Due to the obvious side effects inherent in older-generation anticonvulsants, the two most important anticonvulsants now used for neuropathic pain are gabapentin and pregabalin [34,35]. Pharmacological therapy is considered to have failed if pain persists after pregabalin has been administered for four weeks.

Furthermore, SCB with local anaesthetic agents and with or without steroids can be used to disrupt the afferent pain pathway to relieve CSP. SCB can be used both diagnostically and therapeutically. However, studies have demonstrated that this technique rarely provides long-term relief and often lasts only for the duration of the local anaesthetic. The block is injected by isolating the spermatic cord at the inguinal‒scrotal junction. Then, a 27-gauge needle is used to puncture the spermatic cord at the level of the pubic tubercle. One published recommendation is to use 20 mL of 0.25% bupivacaine hydrochloride for the initial cord block. Most patients experience >90% relief of pain temporarily [19]. Another recommendation is to treat every two weeks until there have been four to five blocks that used 9 mL of 0.75% bupivacaine hydrochloride combined with 1 mL (10 mg) of triamcinolone acetonide. If this treatment does not alleviate pain, repeating the treatment is not recommended [36].

Physical Therapy, Psychotherapy and Other Non-Surgical Options

Up to 10% of patients who present with CSP are found to have musculoskeletal pain localised to areas that may include the conjoint tendon, the adductor tendon and the pelvic floor. These patients may benefit from physical therapies, including myofascial trigger-point release, perineal/pelvic-floor massage or other physiotherapies targeted to these areas, including antibiotics and NSAIDs, as management options [33].

It is well-established that CSP significantly affects one’s quality of life. Also, given the extreme psychological toll associated with chronic pain, there should be a low threshold for involving a mental health professional in the patient’s care plan [22]. Referral to a mental health specialist is indicated if (1) the patient endorses a significant psychiatric response to ongoing pain, (2) the pain affects non-medical aspects of life (relationships, employment, legal issues) or (3) the pain is accompanied by anxiety, depression or significant mental illness [34,35]. Other non-surgical techniques include pulsed radiofrequency of the spermatic cord and the genital branch of the genitofemoral nerve for PVPS if the patient receives temporary relief from an SCB. Acupuncture is rarely mentioned, being reported only in some small non-randomised trials [2,16]. Finally, the least mentioned non-surgical techniques are transcutaneous electrical stimulation (TENS) and vibratory stimulation [16]. Tables 1-4 analyse medical, psychotherapy, physical, SCB, radiofrequency and other non-surgical options of treatment in the last five years.

**Table 1:** Medical treatment NSAID, non-steroidal anti-inflammatory drug; TCA, tricyclic antidepressant; Y, yes

Publications	Management		
	Antibiotics	NSAID	TCA
Gordhan and Sadeghi-Nejad, 2015 [1]	Y	Y	Y
Tan and Levine, 2017 [2]	Y	Y	Y
Calixte et al., 2017 [36]		Y	Y
Calixte et al., 2017 [37]	Y	Y	Y
Tatem and Kovac, 2017 [5]	Y	Y	Y
Wu and Jarvi, 2018 [18]			Y
Wu and Jarvi, 2018 [32]	Y	Y	Y
Levine and Abdelsayed, 2018 [33]	Y	Y	Y
Jarvi et al., 2018 [34]	Y	Y	Y
Ziegelmann et al., 2019 [19]	Y	Y	Y

**Table 2:** Anticonvulsant and psychotherapy management Y, yes

Publications	Management	
	Anticonvulsants	Psychotherapy
Gordhan and Sadeghi-Nejad, 2015 [1]	Y	Y
Tan and Levine, 2017 [2]	Y	Y
Agarwal and Sy, 2017 [35]	Y	
Calixte et al., 2017 [36]	Y	
Tatem and Kovac, 2017 [5]	Y	
WU and Jarvi, 2018 [18]	Y	Y
Wu and Jarvi, 2018 [32]	Y	Y
Levine and Abdelsayed, 2018 [33]	Y	
Jarvi et al., 2018 [34]	Y	Y
Ziegelmann et al., 2019 [19]	Y	Y

**Table 3:** Physical therapy, SCB and radiofrequency management GFN, genitofemoral nerve; SC, spermatic cord; SCB, spermatic cord block; Y, yes

Publications	Management		
	Pelvic floor physical therapy	SCB	Radiofrequency of SC and GFN
Gordhan and Sadeghi-Nejad, 2015 [1]			Y
Tan and Levine, 2017 [2]	Y	Y	Y
Calixte et al., 2017 [36]		Y	Y
Calixte et al., 2017 [37]		Y	
Tatem and Kovac, 2017 [5]	Y	Y	
Wu and Jarvi, 2018 [18]	Y	Y	
Wu and Jarvi, 2018 [32]	Y	Y	
Levine and Abdelsayed, 2018 [33]	Y	Y	
Jarvi et al., 2018 [34]	Y	Y	
Ziegelmann et al., 2019 [19]	Y	Y	Y

**Table 4:** Other options of non-surgical management ENS, transcutaneous electrical stimulation; Y, yes

Publications	Management		
	TENS	Vibratory stimulation	Acupuncture
Tan and Levine, 2017 [2]			Y
Khandwala et al., 2017 [38]		Y	
Jarvi et al., 2018 [34]			Y
Ziegelmann et al., 2019 [19]	Y	Y	Y

Surgical management

Microsurgical Denervation of the Spermatic Cord

Microsurgical denervation of the spermatic cord (MDSC) is a minimally invasive surgical procedure used to manage CSP after the failure of conservative treatments [36,37]. Multiple retrospective studies have examined the use of MDSC for CSP. The success rates for this procedure range from 71% to 96% [34]. Benson et al. conducted a retrospective review of 74 patients who underwent MDSC and found that a positive response to SCB (≥ 50% reduction in pain) predicted pain resolution in 75% of the patients, suggesting that the response to preoperative SCB can predict the success of MDSC [38,39].The risks of the procedure, including persistent pain, persistent numbness, infection, bleeding, testicular atrophy, infertility and hydrocele formation, need to be discussed with the patient as part of informed consent. Given that this procedure involves significant potential complications, it should be performed only in dedicated centres that have expertise in MDSC.

Shiraishi et al. have recommended that high-inguinal MDSC is effective and safe (retaining testicular function) for CSP that is refractory to medical management. They found that the high-inguinal approach is easier than the sub-inguinal approach (used most often) because the former involves fewer divisions of veins and a larger diameter of the spermatic artery [40].

Chaudhari et al. have stated that idiopathic chronic orchialgia remains a difficult condition to manage. If surgery is considered, MDSC should be considered as the first surgical approach to stopping pain while sparing the testicle [41].

The MDSC procedure is a reasonably successful, durable and valuable approach for PVPS, especially when the pain involves multiple structures in the scrotum (testis, epididymis, spermatic cord) [42]. Ultrasound-guided targeted cryoablation (UTC) of the peri-spermatic cord is a safe potential treatment option for salvage management of persistent CSP in patients who have failed MDSC [43].

Surgical treatment includes various modalities; MDSC is an important and minimally invasive surgical procedure for managing CSP after the failure of conservative treatments [36]. It has been noted that MDSC has a superior success rate of not less than 70% [2]. Table 5 analyses the MDSC, UTC and laparoscopic denervation as surgical treatment options in the last five years.

**Table 5:** MDSC, UTC and LD LD, laparoscopic denervation; MDSC, microsurgical targeted denervation of the spermatic cord; UTC, ultrasound-guided cryoablation; Y, yes

Publications	Surgical management		
	MDSC	UTC (after MDSC)	LD
Gordhan and Sadeghi-Nejad, 2015 [1]	Y		Y
Tan and Levine, 2017 [2]	Y		Y
Calixte et al., 2017 [36]	Y	Y	
Calixte et al., 2017 [37]	Y	Y	
Wu and Jarvi, 2018 [32]	Y		
Tan et al., 2018 [42]	Y		
Levine and Abdelsayed, 2018 [33]	Y		
Jarvi et al., 2018 [34]	Y		
Calixte et al., 2019 [43]		Y	
Ziegelmann et al., 2019 [19]	Y		
Shiraishi et al., 2019 [40]	Y		
Chaudhari et al., 2019 [41]	Y		

Vasectomy Reversal, Epididymectomy and Orchidectomy

Vasectomy reversal, epididymectomy and orchidectomy (inguinal approach) are frequently recommended surgical treatments for CSP. The reported success rates of vasectomy reversal range from 34% to 59%, and the rate for epididymectomy ranges from 50 to 92%, with better results reported if a structural abnormality (cyst, granuloma or mass) was noted in the epididymis on examination or ultrasound (US). Orchidectomy can be a treatment of last resort for CSP and has an average success rate of 55.6% [2]. Lowe has concluded that inguinal orchiectomy with high ligation appears to provide the best result for resolving scrotal pain, possibly related to its ability to address the sensory components from the ilioinguinal and genitofemoral nerves [44].

Other Surgical Treatment Options

An increasingly used surgical treatment is Botox injection [34,37] because the botulinum-A toxin has been shown to modulate the release of neuropeptides (substance P and calcitonin gene-related peptide) that inhibit neurogenic inflammation and chronic pain [35]. However, a randomised, double-blind, controlled trial by Dockray et al. found no superiority of onabotulinumtoxinA plus a local anaesthetic compared to a local anaesthetic alone for the control of SCP pain. Interestingly, our open label onabotulinumtoxinA trial noted significant pain improvement, suggesting a potential placebo effect [45].

**Figure 1:**
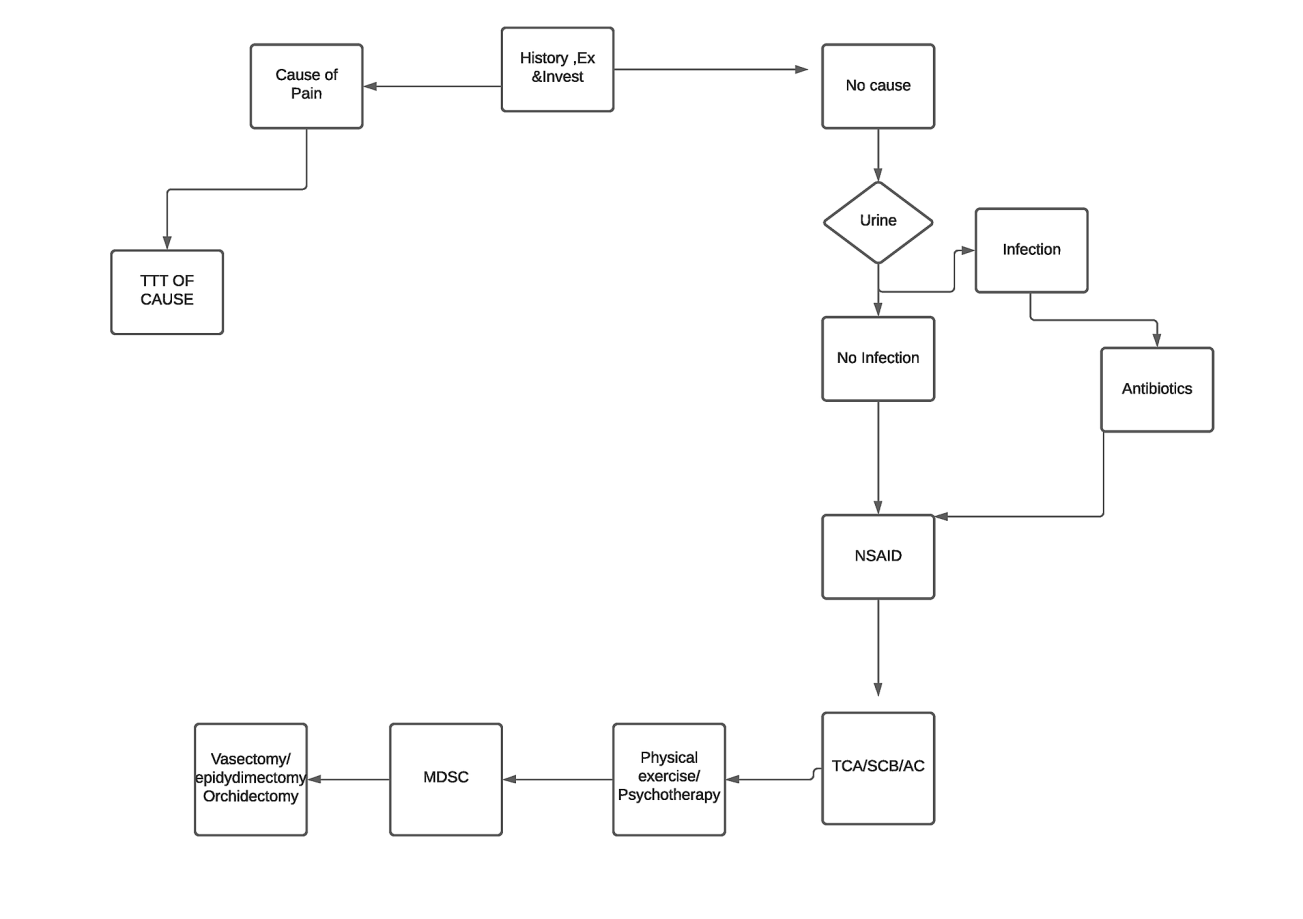
Flow chart suggesting the management strategy of CSP CSP, chronic scrotal pain

Other, rarely mentioned, surgical treatments for CSP that need more research include laparoscopic denervation [1,2], robotic denervation [46], resection of the genitofemoral nerve [2], sperm granuloma resection [16], AmnioFix® injection [36,37], spinal cord stimulation [36], scrotoscopy using the novel autoclave [47] and microsurgical sub-inguinal cremaster muscle release (MSCMR), which has been shown to be an effective option that has a relatively low rate of complications in cases of chronic orchialgia that has been clearly identified as secondary to testicular retraction due to a hyperactive cremaster muscle reflex and in which all other aetiologies of orchialgia have been ruled out [48]. Table 6 analyses vasectomy reversal, epididymectomy and orchidectomy in the last five years’ publications. Figure 1 shows a flow chart of recommended CSP management.

**Table 6:** Vasectomy reversal, epididymectomy and orchidectomy Y, yes

Publications	Surgical management		
	Vasectomy reversal	Epididymectomy	Orchidectomy
Gordhan and Sadeghi-Nejad, 2015 [1]	Y	Y	Y
Tan and Levine, 2017 [2]	Y	Y	Y
Tatem and Kovac, 2017 [5]	Y		Y
Calixte et al., 2017 [36]	Y	Y	Y
Calixte et al., 2017 [37]	Y		Y
Lowe, 2017 [44]			Y
Wu and Jarvi, 2018 [32]	Y	Y	Y
Levine and Abdelsayed, 2018 [33]	Y	Y	Y
Jarvi et al., 2018 [34]	Y	Y	Y
Ziegelmann et al., 2019 [19]	Y	Y	Y

## Conclusions

CSP remains a topic on which the available evidence is sparse and of low quality, making it difficult to strongly recommend individual treatment options. However, it is recommended to review each case in detail, which includes taking detailed histories, especially medical and social ones, conducting examinations, of which abdominal and DRE ones are the most crucial, and employing diagnostic modalities (urinalysis is crucial and US is helpful). Treatment options include medical treatment, physical therapy and psychotherapy, all of which may help patients and provide useful tools for coping with this condition. Depending on the patient’s state, the severity of the complaint and what options have already been tried, there are also useful surgical options for CSP, with MDSC being the one most recommended.
